# Validating α-particle emission from ^211^At-labeled antibodies in single cells for cancer radioimmunotherapy using CR-39 plastic nuclear track detectors

**DOI:** 10.1371/journal.pone.0178472

**Published:** 2017-06-28

**Authors:** Satoshi Kodaira, Huizi Keiko Li, Teruaki Konishi, Hisashi Kitamura, Mieko Kurano, Sumitaka Hasegawa

**Affiliations:** 1 Radiation Measurement Research Team, National Institute of Radiological Sciences, National Institutes for Quantum and Radiological Science and Technology, Chiba, Japan; 2 Radiation and Cancer Biology Team, National Institute of Radiological Sciences, National Institutes for Quantum and Radiological Science and Technology, Chiba, Japan; 3 JSPS Research Fellow, Graduate School of Medical and Pharmaceutical Sciences, Chiba University, Chiba, Japan; 4 Regenerative Therapy Research Team, National Institute of Radiological Sciences, National Institutes for Quantum and Radiological Science and Technology, Chiba, Japan; German Cancer Research Center (DKFZ), GERMANY

## Abstract

Recently, ^211^At has received increasing attention as a potential radionuclide for cancer radioimmunotherapy. It is a α-particle emitter, which is extremely effective against malignant cells. We demonstrate a method to verify the efficiency of ^211^At-labeled trastuzumab antibodies (^211^At-trastuzumab) against HER2 antigens, which has not been determined for radioimmunotherapy. A CR-39 plastic nuclear detector is used for measuring the position and the linear energy transfer (LET) of individual ^211^At α- particle tracks. The tracks and ^211^At-trastuzumab-binding cells were co-visualized by using the geometric information recorded on the CR-39. HER2-positive human gastric cancer cells (NCI-N87), labelled with ^211^At-trastuzumab, were dropped on the centre of the CR-39 plate. Microscope images of the cells and the corresponding α-tracks acquired by position matching were obtained. In addition, 3.5 cm × 3.5 cm macroscopic images of the whole plate were acquired. The distribution of number of α-particles emitted from single cells suggests that 80% of the ^211^At-trastuzumab-binding cells emitted α-particles. It also indicates that the α-particles may strike the cells several times along their path. The track-averaged LET of the α-particles is evaluated to be 131 keV/μm. These results will enable quantitative evaluation of delivered doses to target cells, and will be useful for the *in vitro* assessment of ^211^At-based radioimmunotherapeutic agents.

## Introduction

The radionuclide ^211^At is a promising candidate for targeted radioimmunotherapy because of several advantages. It is a high linear energy transfer (LET) α-emitter that deposits enough energy (>80 keV/μm) to break double-stranded DNA [1]. It has a short emission range (<50 μm) inside cells, and has a 7.2-hr treatment half-life [2]. Moreover, the decay sequence of ^211^At (α-decay to ^207^Bi, or electron capture leading to ^211^Po alpha-decay to ^207^Pb) has reduced side effects from daughter nuclei because of the short ^211^Po half-life (0.5 sec); this more favourable that the ^210^At decay sequence, which is another candidate for targeted radionuclide therapy. Because of its high LET, ^211^At is expected to be very toxic to cells [1,3–5]. However, the LET distribution of emitted α-particles from ^211^At-binding cells has not been investigated.

Targeted radioimmunotherapy uses an antibody labelled with a cytotoxic radionuclide to selectively deliver radiation to target cancer cells [6,7]. The therapeutic application of ^211^At-labeled monoclonal antibodies or antibody fragments for cancer radioimmunotherapy has been actively investigated. In preclinical models, ^211^At-labeled monoclonal antibodies were effective against lymphoma and various solid tumours [8–10]. Phase I studies of ^211^At-labeled MX35 F(ab’)2 in ovarian cancer patients were conducted to investigate toxicity and to determine the pharmacokinetics for evaluating absorbed doses to normal tissues [11]. These studies verified antibody labelling of ^211^At and demonstrated that it could become a potential therapeutic option.

Given the successful results of the ^211^At preclinical trials, the mechanistic aspects still need to be understood to strengthen the models. For example, the estimated radiation dose within the targeted cell/area is important information for characterizing the effectiveness of the labelled antibodies. Furthermore, the binding efficiency of the antibody on the targeted protein/cell depends on various biological factors and would directly influence the total doses. Thus, the number of α-particle traversals per individual cell is essential information, especially for *in vitro* studies of mammalian cells, because the high LET of α-particles would considerably change the total dose and the fate of the irradiated cells. However, a methodology has not been established to provide these physical parameters at the single-cell level.

CR-39 plastic nuclear track detectors are promising tools not only for LET measurements and for determining α-particle doses in a single cell, but also for autoradiographic imaging of the spatial dose distribution in the tissue [12,13]. This is because the dosimetry is based on LET spectroscopy [14,15], and the autoradiography [12,13] has sub-micron position resolution under optical microscopy. Several co-visualization methods of cells and ion tracks have been developed with CR-39. Cells cultured on a CR-39 plate that is irradiated with heavy ions enable the visualization of both ion traverses and cellular locations [16]. Slices of ^10^B-labeled mice organs that have been mounted on a CR-39 plate and irradiated with thermal neutrons enable the visualization of the ^10^B concentration because of ^10^B(n,α)^6^Li neutron capture in the organ/tumour [13,17,18].

Here, we examine α-particle emission in single cells from ^211^At-trastuzumab antibodies that target HER2 proteins, by using a CR-39 plastic nuclear track detector. This will be useful for *in vitro* assessment of ^211^At-based radioimmunotherapeutic agents for target cells.

## Materials and methods

### Principle of ion track detection

An energetic ion passing through CR-39 makes a trail of radiation damage (or latent track) along its trajectory [19]. A chemical etchant preferentially removes the latent track with a track etch rate *V*_t_, and removes undamaged regions with a bulk etch rate *V*_b_. As a result, a conical track (or etch pit) appears in the detector. The track registration sensitivity *S* is defined as the reduced etch rate (*S*≡*V*_t_/*V*_b_-1). Its value is scaled as a function of the restricted energy loss (REL) in CR-39, which takes into account δ-ray contributions in track registration [20]. The REL value of an ion is obtained from the measured *S* value by using the response curve between *S* and REL, calibrated with heavy ion beams [15]. The 200-eV δ-ray cut-off energy has been empirically determined for CR-39 [21]. The REL value for CR-39 [MeV·cm^2^/g] can be converted into LET in water *L* [keV/μm] by using a conversion function of LET/REL [22]. The track fluence *F* [cm^−2^] in each LET bin (Δ*L*) yields the LET spectrum.

*S* is obtained from the geometrical parameters of the etch pit:

$$S\equiv \frac{V_{t}}{V_{b}}-1=\sqrt{\frac{16B^{2}D_{A}^{2}}{{\left(4B^{2}-D_{B}^{2}\right)}^{2}}+1}-1.$$ (1)

Here, *D*_A_ and *D*_B_ are the major and minor axes of the elliptical etch pit, respectively, and *B* is the amount of bulk etching (*B* = *V*_b·_*t*, where *t* is the etching time).

The detectable incident angle in the detector is limited by *S* (*i*.*e*., LET), because of the critical angle of incidence [23]. A track with an incident angle larger than the critical angle is not observed because the thickness of the layer removed by chemical etching at rate *V*_b_ is equal to or greater than the track length produced by the rate *V*_t_. A charged particle can form an etchable track at the incident angle between π/2 and π/2 –*θ*_c_, where *θ*_c_ is the critical incident angle. The *θ*_c_ is defined as,

$$\theta_{c}={cos}^{-1}\left(\frac{1}{1+S}\right).$$ (2)

The detection efficiency (*E* [track/particle]) is calculated by,

$$E=\frac{\int_{\theta_{c}}^{\pi /2}\sin\theta \cos\theta d\theta }{\int_{0}^{\pi /2}\sin\theta \cos\theta d\theta }=1-\sin^{2}\theta_{c}=\frac{{\left(S+1\right)}^{2}-1}{{\left(S+1\right)}^{2}}.$$ (3)

Therefore, the total fluence ($\bar{N}(L)$) is obtained from the measured fluence (*N*(*L*)) depending on *L* by,

$$\bar{N}(L)=\frac{N(L)}{E(L)}.$$ (4)

To accurately measure the LET spectrum, it is necessary to properly correct for this angular sensitivity in the measurements. The detailed procedure for solid angular correction is described elsewhere [24, 25].

A 50 mm × 50 mm × 0.9 mm CR-39 plate (HARZLAS/TD-1, Fukuvi Chemical Industry, Japan) was used. HARZLAS/TD-1 is sensitive to energetic ions with LET in water that ranges over 3.5−420 keV/μm [15]. The detector response was calibrated using various heavy ions with known charge, energy, and LET that were produced at the Heavy Ion Medical Accelerator in Chiba. The LET of observed particle in CR-39 is obtained from the measured *S* by using the calibration curve [15].

### Experimental

The HER2-over-expressing human gastric cancer cell line NCI-N87 (N87) was obtained from the American Type Culture Collection (Manassas, VA). The cells were maintained in RPMI 1640 medium supplemented with 10% fetal bovine serum (, Nichirei Biosciences, Tokyo, Japan), 100 μg/ml penicillin, and 100 μg/ml streptomycin in a 37°C humidified atmosphere containing 5% CO_2_.

Trasutuzumab is a humanized anti-HER2 monoclonal antibody that was purchased from Chugai Pharmaceutical (Tokyo, Japan). ^211^At was produced at the National Institute of Radiological Sciences AVF-930 cyclotron (Chiba, Japan) [26]. The ^211^At-labeled trastuzumab (^211^At-trastuzumab) [27] was isolated with size exclusion chromatography using a Sephadex 50 spin column (GE Healthcare UK Ltd., Little Chalfont, England) and phosphate-buffered saline (PBS) at room temperature (730 g, 2 min).

Cell suspensions (5×10^6^ cells/ml) were prepared in 1% BSA in PBS. The cells were incubated for one hour with ^211^At-trastuzumab (3.565 kBq, 42.24 ng/ml) in a 2 ml tube on ice and were mixed every 15 minutes. The suspension was then centrifuged (114 g, 5 min at 4°C) and washed twice with cell suspension buffer to remove unbound ^211^At-trastuzumab and ^211^At in the supernatant. The supernatant was removed and 5 ml of ice-cold methanol was gently added for fixation.

**Fig 1:**
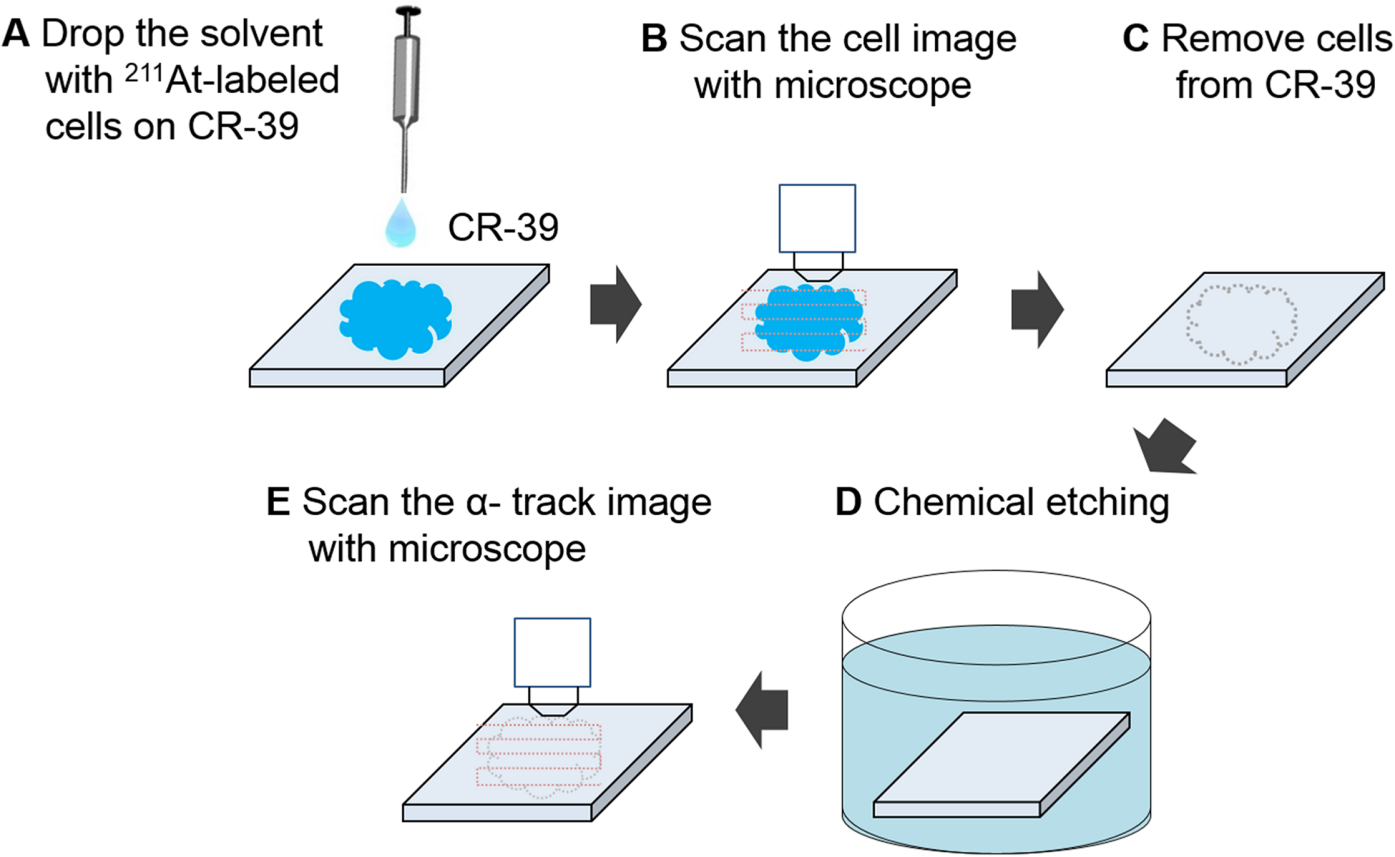
Schematic of the experimental procedure.

The following experimental procedures are illustrated in Fig 1. (A) A 10-μl drop containing 1×10^4^ cells was placed at the centre of a CR-39 plate. The solvent immediately spread out to approximately 20 mm in diameter. (B) Images of the cells were acquired with a wide-area optical microscope equipped with a 20×, NA0.45 objective lens (FlexScope, Seiko Precision Inc., Japan). The image size was 9.43 cm^2^ and the pixel size was 0.28 μm/pixel. (C) After 14 hours, corresponding to two half-lives duration of ^211^At decay, the cells were removed from the CR-39 surface with wipes (Kimwipes) impregnated with ethanol. (D) The CR-39 substrate was then etched in 7M NaOH at 70°C for 2 hours. (E) Microscope images of α-particle tracks in the CR-39 were acquired under the same conditions noted in (B).

Images were analyzed with PitFit software developed for analyzing etched pits in CR-39 [28]. It analyzes their size and position (*x*, *y*) on the CR-39 coordinates, and the α-particle track size is determined in terms of *D*_A_ and *D*_B_ by a least-squares ellipsoidal fit. The software was also used to analyse the *X*-*Y* coordinates of each cell to compare their geometric distribution with the α-particle tracks, as described below.

The coordinates on the CR-39 plate before and after etching were adjusted and corrected by the Affine transformation technique using locations scratched with a small diamond pen before the experiments. The track coordinate (*X*_track_, *Y*_track_) is related to be the corresponding cell coordinate (*X*_cell_, *Y*_cell_) using affine parameters (*a*, *b*, *c*, *d*, *p* and *q*) by:

$$\left(\begin{array}{c} X_{cell}\\ Y_{cell} \end{array}\right)=\left(\begin{array}{cc} a & b\\ c & d \end{array}\right)\left(\begin{array}{c} X_{track}\\ Y_{track} \end{array}\right)+\left(\begin{array}{c} p\\ q \end{array}\right).$$ (5)

The affine parameters determined from three landmark points were *a* = 1.001, *b* = 0.002, *c* = -0.002, *d* = 1.003, *p* = -11.057 and *q* = -28.929, respectively. Thus, the positions of α-tracks were then corrected and matched with the positions of cells. The mean distance ($\sqrt{{\left(X_{cell}-X_{track}\right)}^{2}+{\left(Y_{cell}-Y_{track}\right)}^{2}}$) between cell position and transformed track position was found to be 10.1 μm, which would be within almost cell diameter (~10 μm). This technique is frequently used for reconstruction of particle trajectories in CR-39 stacks to characterize nuclear fragmentation reactions of heavy ions with matter [29–31].

The amount of CR-39 bulk etching *B* (2.8±0.3 μm) was determined from the change of weight of detector before and after etching [32]. The value of *S* was obtained from Eq (1).

## Results and discussion

### Autoradiographs

**Fig 2:**
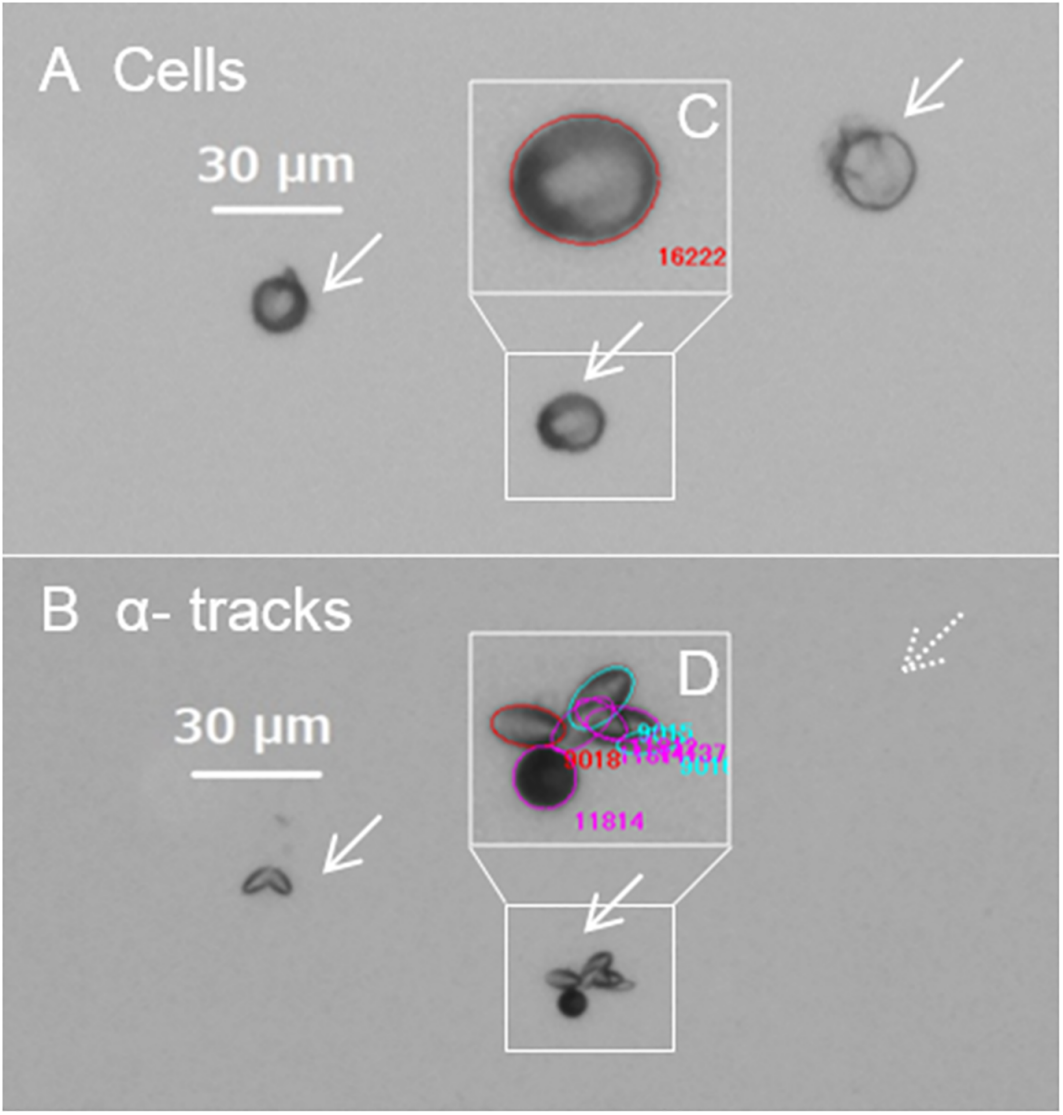
Microscope images of cells and emitted α-tracks from those cells on aCR-39 detector. A) Cells and B) corresponding α-tracks. A dot arrow indicates that no α-tracks are observed in the corresponding cell position. The enlarged images of C) cells and D) α-tracks recognized by ellipse fitting result are inset by the colored circles.

Typical microscopic images of cells and their emitted α-tracks are shown in Fig 2A and 2B, respectively. The three isolated single cells in Fig 2A correspond to the clusters of α-particle tracks, 2 tracks, 7 tracks and 0 tracks from the left in Fig 2B. The case of 0 tracks (indicating by a dot arrow) means that no α-tracks are observed in the corresponding cell position. The enlarged microscopic images of the cells and α-particle tracks recognized by ellipse fitting are inset as shown in Fig 2C and 2D. The cells look to be shrunken because of the methanol position-fixing on the CR-39 surface. It should be remarked that there is a potential of missing tracks due to the overlap tracks in case of high dense track concentration. In this experiment, we did not observe such α-tracks cluster, which is too dense to miss the significant number of tracks.

**Fig 3:**
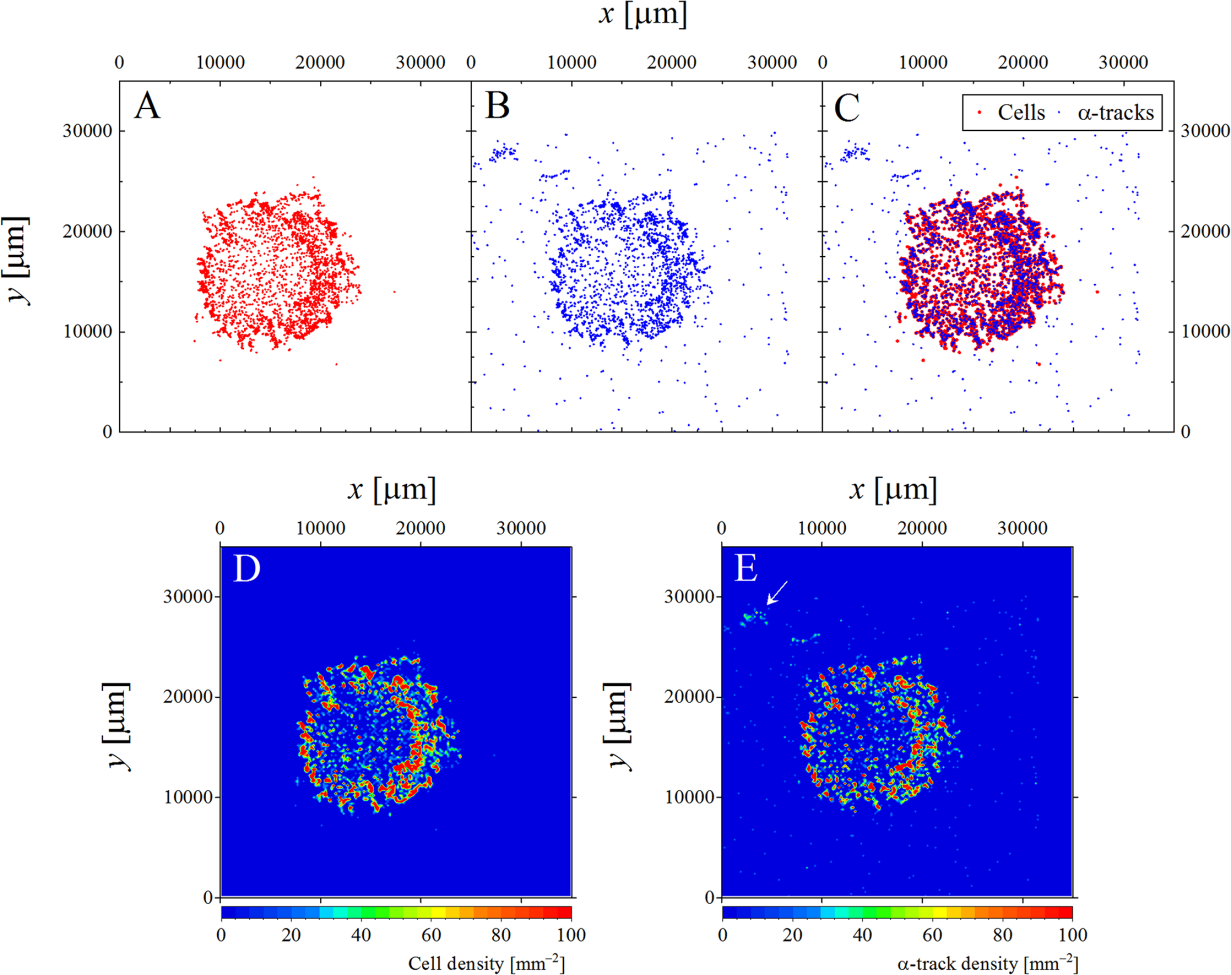
Scatter plots and contour maps of cells and α-tracks positions. Scatter plots of the positions of A) cells and B) α-tracks over the entire surface of the CR-39 detector and C) their overlay, respectively. The macroscopic autoradiography as a contour map by counting the number of D) cells and E) α-tracks in binned positions (Δ*x*, Δ*y*) with 200 μm intervals.

Meanwhile, Fig 3A, 3B and 3C illustrate the scatter plots of the positions of individual cells and tracks over the entire CR-39 surface and their overlay, respectively. The individual cells and α-tracks are obviously correlated in a cm-scale order. These are visualized by counting the number of cells/tracks in binned positions (Δ*x*, Δ*y*) with 200 μm intervals. Fig 3D and 3E demonstrate the macroscopic autoradiography as a contour map of dose distribution in a tissue size order. The structure of the spread-out methanol solvent containing the ^211^At-binding cells is clearly observed. The distribution of α-tracks is consistent with the cell distribution, and includes those from background radionuclides such as radon and thoron. The contribution from the background was estimated from analysis of the opposite (reverse side) surface of the CR-39; it was 132 ± 11 tracks, which is about 2% of the total number of α-tracks (5671 ± 75). The error is statistical. Tracks observed outside of the main part of the dropped solution (pointed by an arrow in Fig 3B) are inconsistent with the cell distribution in Fig 3A. This may be because of residual ^211^At-trastuzumab that was not removed. It is supposed to be due to the floating ^211^At non-binding with cells in the solution, which is specific issue with chemicals on this vitro experiment. The number of α-tracks due to the floating ^211^At non-binding with cells was found to be 353 ± 19 tracks in the whole analyzed area on CR-39, which includes the natural background as mentioned above. The floating ^211^At makes also the cluster of α-tracks as well as binding with a cell. The fluence of floating tracks was obtained to be 3.74 × 10^−7^ [tracks/μm^2^], which would be randomly appeared on the whole analyzed region.

Thus, CR-39 not only enables microscopic imaging of α-tracks, but also allows whole sample macroscopic autoradiographs. Furthermore, this technique should be applicable for imaging micro/macroscopic α-particle distributions of tissue slices of small laboratory animals injected with ^211^At-labeled agents.

### Efficiency of α-particle emission

**Fig 4:**
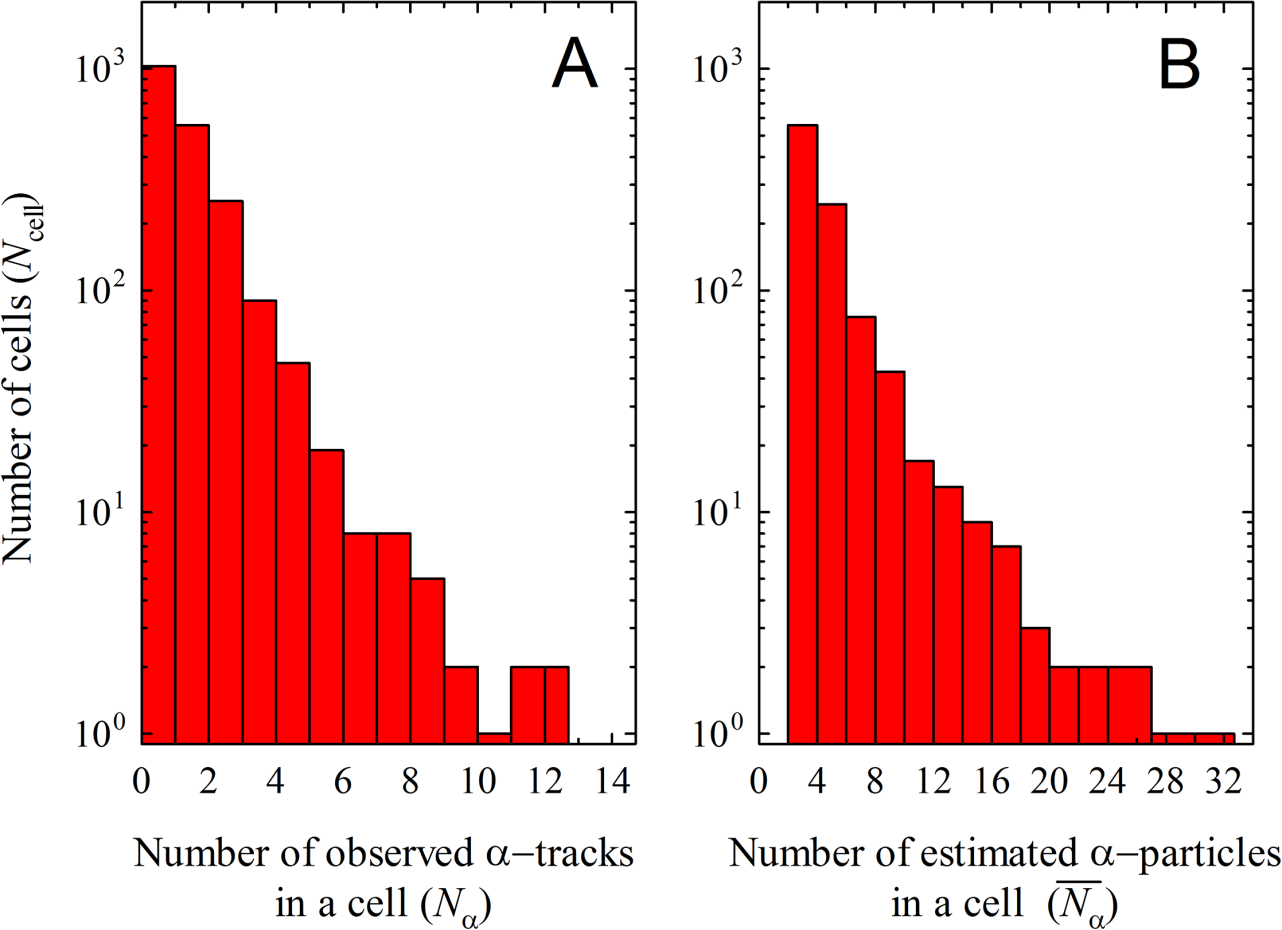
The number of cells *N*_cell_ vs. α-tracks (*N*_α_) and α-particles ($\bar{N_{\alpha }}$). A) The observed number of α-tracks (*N*_α_) emitted from single cells and B) the estimated number of α-particles ($\bar{N_{\alpha }}$) corrected with solid angular and critical angular dependencies.

As seen in Fig 2A and 2B, the number of α-tracks is measurable at the single-cell level. We have selected only isolated single cells for avoiding the ambiguity due to neighboring cells. The number of α-tracks emitted from a single cell was analyed for 2023 cells. Fig 4A plots the number of cells *N*_cell_ *vs*. the number of α-tracks *N*_α_ emitted from those cells. The value of *N*_cell_ decreases with increasing *N*_α_, and the maximum was *N*_α_ = 12 ± 3 from a single cell. The number of α-particles originating in a cell ($\bar{N_{\alpha }}$) is estimated by taking correction of solid angular and critical angular dependencies in the detector [24,25] as shown in Fig 4B. The cell emits α-particles from $\bar{N_{\alpha }}$ = 2 to 32, while no cells are appeared in $\bar{N_{\alpha }}$ = 0 and 1. It is observed that the half of the ^211^At-trastuzumab-binding cells emitted α-particles with *N*_α_ ≥1 (993 cells, 49% in total number of cells). It must be remarked that this data is a record of observed α-tracks from only one side of the CR-39 detector (that which supported the cells). Because the α-particles are emitted in a 4π direction without angular modification during the decay mode, *N*_α_ = 0 data must include *N*_α_ = 1 case which emits to the opposite direction for CR-39 surface supporting the cells. If the emission distribution is isotropic, the same amount of observed *N*_α_ = 1 (993 cells) would emit to the opposite direction for CR-39 surface that is not recorded. Therefore, *N*_α_ = 1 (observed number of tracks without corrections) in a cell will be corrected to be $\bar{N_{\alpha }}$ = 2 at least. Furthermore, *N*_α_ = 0 must include still missing events due to the critical angular dependency (i.e. invisible angular region depending on LET due to the detector acceptance) and truly having none events. Because of difficulty in the experimental identification of these pathways, we have computed the missing probability due to the critical angular dependency in the current LET range using Eq (2). As a result, it is assumed that the observed number of α-tracks would have 21% missing probability due to the critical angular dependency. Now, *N*_α_ ≥1 occupies 993 cells in a total (2023 cells), so that *N*_α_ = 0 (1030 cells) will consist of 515 cells due to the missing by solid angular dependency, 108 cells due to the missing by critical angular dependency, and residual 407 cells truly having none event. Therefore, it is suggested that 80% (= (993+515+108)/2023×100%) of cells would emitα-particles with *N*_α_ ≥1. In other words, the binding efficiency *η* of ^211^At with cells is estimated to be 80%. It should be also noted that the number of emitted α-particles would be slightly increased after the two half-lives employed in this work, which may affect the result of binding efficiency.

The contribution due to the α-tracks from ^211^At non-binding with cells (3.74 × 10^−7^ [tracks/μm^2^]) was negligibly estimated to be less than 0.1 tracks in the total area of analyzed single cells (1.59 × 10^5^ [μm^2^]), if we assume the typical cell radius of 5 μm [33]. It should be noted that the clusters from floating ^211^At may give unwanted dose in normal cells from the viewpoint of given dose, so that the binding efficiency will be an important parameter for the radioimmunotherapy.

Thus estimated binding efficiency may be affected by multiple steps in the protocol, such as the immuno-staining incubation, repeated centrifugation, and fixation. The results may not be relevant for *in vivo* experiments. However, this efficiency is an essential parameter for evaluating locally delivered doses and directly relates to that for cell toxicity of the employed agents.

The expected range in water of a α-particle from ^211^At is about 50 μm; thus, it will hit cells several times along its passage. If there are *N*_cell_ cells with radius *r* (μm) are located in a sphere with radius *R* (μm) corresponding to the α-particle range, where

$$N_{cell}\leq {\left(\frac{R}{r}\right)}^{3}.$$ (6)

then the effective volume [μm^3^] of α-particles hitting cells is:

$$\pi {\left(\sigma +r\right)}^{2}\times R\times \bar{N_{\alpha }}\times n_{cell},$$ (7)

The value σ is a track core radius of a α-particle, $\bar{N_{\alpha }}$ is the number of α-particles emitted from a single cell, and *n*_cell_ is the number of cells emitting $\bar{N_{\alpha }}$ α-particles. Here, σ is small relative to *r*; thus (σ+*r*)≈*r*. The total volume [μm^3^] of cells in the sphere is:

$$\frac{4}{3}\pi r^{3}\times N_{cell}.$$ (8)

Hence, the fraction *f* of α-particles that hit cells is obtained by dividing Eq (7) by Eq (8):

$$f=\frac{3}{4}\left(\frac{R}{r}\right)\bar{N_{\alpha }}\left(\frac{n_{cell}}{N_{cell}}\right).$$ (9)

In Eq (9), the last term corresponds to the binding efficiency *η*. If we assume that *R* = 50 μm, *r* = 5 μm (a typical cell radius [33]), $\bar{N_{\alpha }}=2$ and *η* = 0.80 (i.e. 80%), then *f* = 12. This simple model result suggests that one α-particle passes through several cells, i.e. a cell is hit ten times or more by α-particles.

### LET spectrum and track-averaged LET

**Fig 5:**
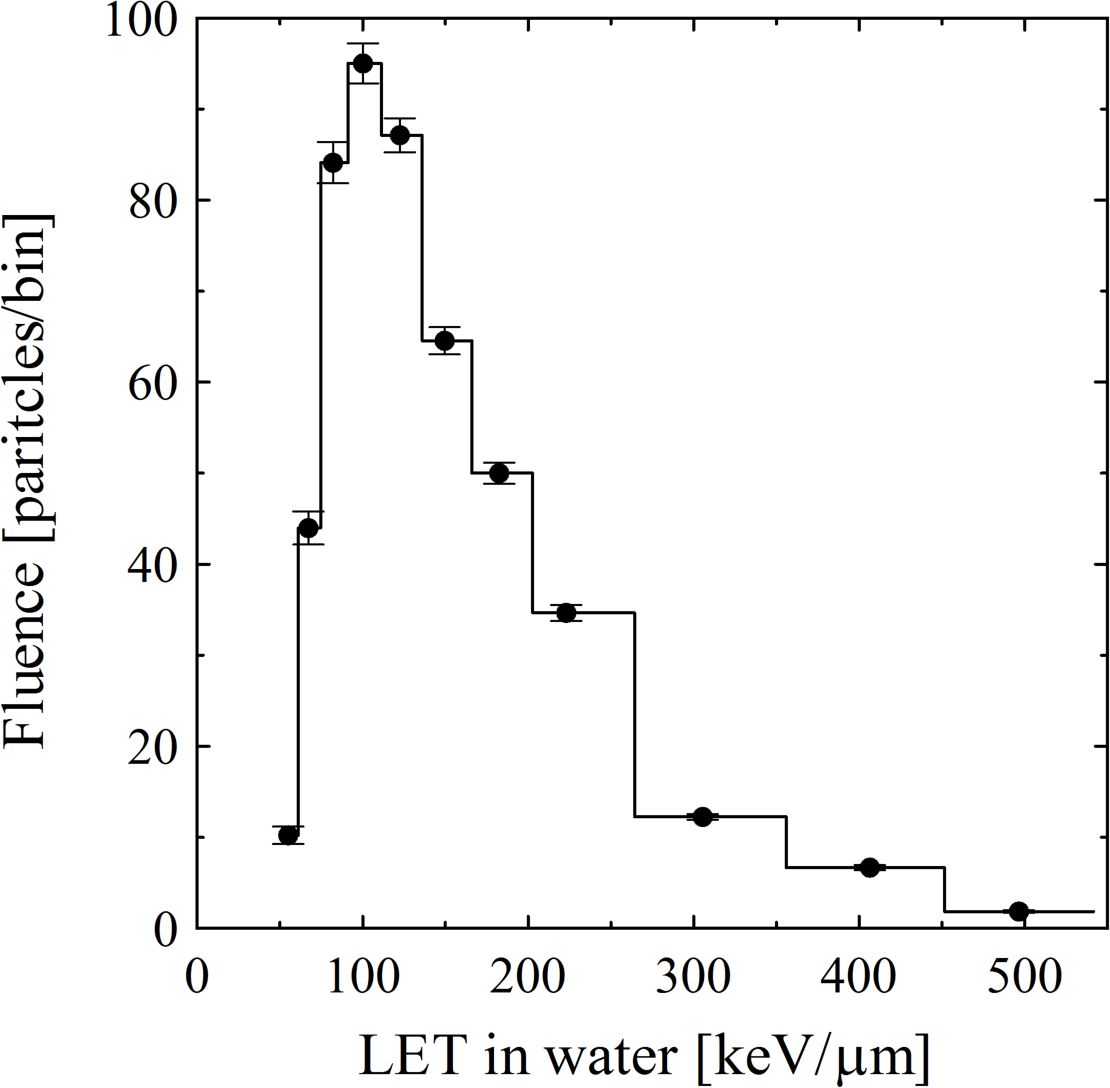
LET spectrum of α-particles from single cells.

CR-39 provides not only position information but also LET dosimetry of individual α-tracks. Fig 5 shows the LET spectrum of α-particles emitted from single cells, as discussed in Fig 4B. Here, the fluence in each LET bin is corrected for solid angles by considering the solid angular dependency related to intrinsic CR-39 features [24,25]. The LET spectrum has a peak around 100 keV/μm for the distribution over 50–500 keV/μm. The expected LET value of 5.72 MeV α-particles from ^211^At decay is 81 keV/μm. The high LET component of >81 keV/μm may be explained by α-particles that lose their energy passing through cells. The low LET component of <81 keV/μm may be explained by high energy 7.45 MeV α-particles emitted from ^211^Po (LET: 69 keV/μm) originating from ^211^At electron-capture with a 58% branching ratio. Another explanation is related to LET resolution in the CR-39 detector, which basically has better resolution for high LET particles such as 2% for heavy particles with LET~100 keV/μm [15]. The resolution is, however, dependent on the amount of bulk etch *B* [34]. In general, the decrease of B corresponds to the decrease of track size, resulting that the measurement accuracy of the track becomes relatively low against the optical microscopic resolution. We assume that the LET resolution as a systematic error is about 18% (1σ) in the condition of *B* = 2.8 μm and LET~100 keV/μm, according to previous reports [15, 34].

The track-averaged or fluence-averaged LET [35,36] will be applicable for evaluating the mean value for emitted α-particles from cells. The track-averaged LET *L*_t_ is defined as the mean LET in water *L* weighted by track fluence *F* in each LET bin Δ*L*:

$$L_{t}=\frac{\sum L\times F\times \Delta L}{\sum F\times \Delta L}.$$ (10)

The *L*_t_ value of α-tracks emitted from ^211^At-labeled agents was evaluated to be 131 ± 5 (statistical) ± 24 (systematic) keV/μm.

### Delivered dose

The number of α-particles in a single cell $\bar{N_{\alpha }}$ and the track-averaged LET *L*_t_ yield a delivered absorbed dose *D*_cell_ [Gy] in a single cell:

$$D_{cell}=\frac{1.6\times 10^{-9}}{\rho }\times \frac{\bar{N_{\alpha }}}{<A>}\times L_{t},$$ (11)

where ρ = 1.0 (g/cm^3^) is assumed as the density of water, <*A*> (cm^-2^) is the average cross-section of a single cell. If we assume a typical mean cell radius of 5 μm [33], then *D*_cell_ [Gy] is expressed as:

$$D_{cell}\approx 0.3\;\bar{N_{\alpha }}.$$ (12)

The relationship between *D*_cell_ and cellular toxicity will yield the minimum $\bar{N_{\alpha }}$ for cell death. There are many previous reports [4,5] that describe the toxicity of α-particles for the energy range 3.26–5.6 MeV, which is similar to the present work. Multiple traversals of α-particles are necessary for cell inactivation, and the number of hits for cell death would depend on the cell line and the energy deposition of the α-particles. Thus, this method enables the estimation of important factors such as the number of tracks, as well as the cancer therapy dose for targeted cells/tissues. Further examination of cellular lethality as a result of α-particles from ^211^At will be required for evaluating a delivered dose.

## Conclusion

We used CR-39 plastic nuclear track detectors for measuring α-particle tracks emitted from ^211^At-trastuzumab in single cells. Optical microscope images of cells on the CR-39 plate and corresponding emitted α-tracks matched by position were obtained. In addition, macroscopic images of the entire CR-39 plate were obtained. The distribution of number of α-particle emitted from a single cell suggests that 80% of the ^211^At-trastuzumab-binding cells emitted α-particles. This indicates that the α-particles may hit cells several times along their passages within their ranges. The track-averaged LET of α-particles was evaluated to be 131 keV/μm. These efficiencies of α-particle emission from ^211^At and the track-averaged LET of α-particles will allow quantitative evaluation of delivered doses to target cells. The CR-39 technique described here provides a useful *in vitro* assessment of ^211^At-trastuzumab for targeting cells, and will contribute to the development of ^211^At-labeled monoclonal antibodies for cancer radioimmunotherapy.
